# Supplementary material for: The transcription factor Zfh1 acts as a wing-morph switch in planthoppers
Source: Nat Commun. 2022 Sep 27;13:5670. doi: 10.1038/s41467-022-33422-6 (PMC9515195; doi:10.1038/s41467-022-33422-6)
Supplement: Supplementary file 1 — Supplementary Information [file 41467_2022_33422_MOESM1_ESM.pdf]

The transcription factor Zfh1 acts as a wing-morph switch in planthoppers

Jin-Li Zhang, Sun-Jie Chen, Xin-Yang Liu, Armin P. Moczek, Hai-Jun Xu

This Supplementary information includes:

Supplementary Figs. 1-7

Supplementary Table 1

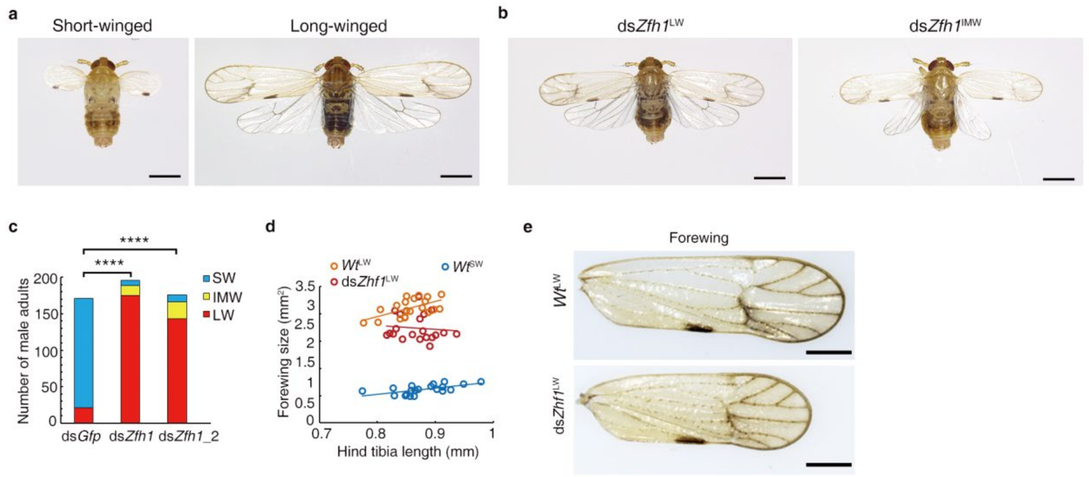

Supplementary Fig. 1. Knockdown of *Zfh1* leads to long-winged males. a Wild-type long-winged (LW) and short-winged (SW) males. **b** Morphology of ds*Zfh1*^{LW} and ds*Zfh1*^{IMW} males. ds*Zfh1*^{LW} and ds*Zfh1*^{IMW}, ds*Zfh1*-treated BPHs with long and intermediate-size (IMW) wings, respectively. **c** Number of males with different wing morphs upon dsRNA treatments. The LW ratio is compared between two columns using Pearson χ^2 test (**** P = 2.9851E-49, χ^2 = 217.623 and df = 1 for ds*Zfh1* vs ds*Gfp*; **** P = 7.0364E-38, χ^2 = 165.522 and df = 1 for ds*Zfh1*_2 vs ds*Gfp*). **d** Wing size and hind tibia length in ds*Zfh1*^{LW}, *Wt*^{LW} and *Wt*^{SW} males. Each circle represents a single male (n = 20). **e** Vein patterning on forewings from *Wt*^{LW} and ds*Zfh1*^{LW} males. Twenty samples were repeated independently with similar results. Source data are provided as a Source Data file.

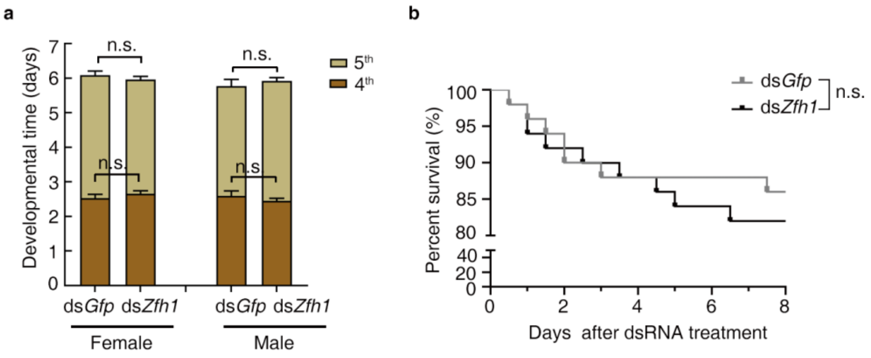

Supplementary Fig. 2. Developmental duration and survival rates of nymphs upon dsRNA treatments. a Duration of fourth- and fifth-instar nymphs treated with ds*Zfh1* and ds*Gfp*. Third-instar *Wt*^{SW} nymphs were microinjected with ds*Zfh1* or ds*Gfp*. Bars (mean ± s.e.m.) are derived from individual BPHs (ds*Zfh1*, n = 13 females and n = 19 males; ds*Gfp*, n = 17 females and n = 11 males). Two corresponding columns are compared using two-tailed unpaired *t*-test (n.s., non-significant). **b** Survival rates of ds*Zfh1* or ds*Gfp* nymphs. Third-instar *Wt*^{SW} nymphs were microinjected with ds*Zfh1* (n = 50) or ds*Gfp* (n = 50) and surviving nymphs are monitored every 12 h. Log-rank Mantel-Cox test (n.s., non-significant, χ^2 = 0.2753, and df = 1). Source data are provided as a Source Data file.

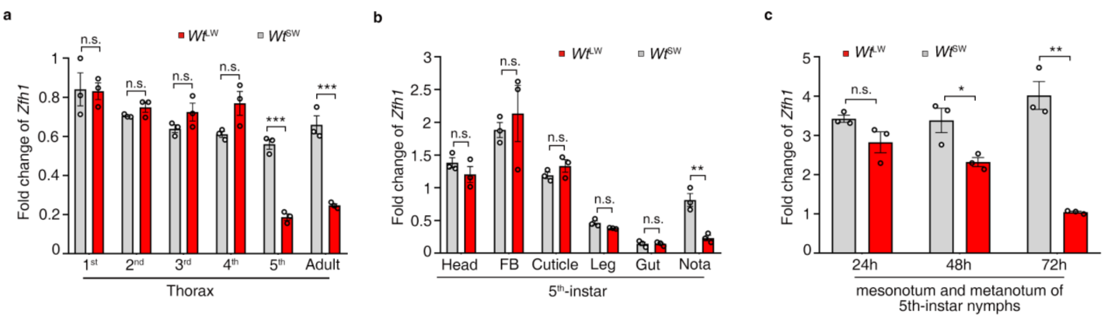

Supplementary Fig. 3. The spatiotemporal expression of *Zfh1* in wild-type short-winged (*Wt*^{SW}) and long-winged (*Wt*^{LW}) BPHs. a The temporal expression of *Zfh1* in the thorax of *Wt*^{SW} and *Wt*^{LW} BPHs across developmental stages. **b** Tissue-specific expression of *Zfh1* in fifth-instar *Wt*^{SW} and *Wt*^{LW} nymphs. Nota represent mesonotum and metanotum. FB, fat body. **c** The temporal expression of *Zfh1* in mesonotum and metanotum of *Wt*^{SW} and *Wt*^{LW} nymphs during the fifth-instar stage. The relative expression of *Zfh1* is normalized to the expression level of *rps11* in qRT-PCR assay. The experiments were repeated three times with similar results (circles). Data are presented as mean ± SEM. Two-tailed unpaired *t*-test was used for the statistical analysis (**a**, *** $P = 0.000247$ for 5th and *** $P = 0.000909$ for adult; **b**, ** $P = 0.005627$; **c**, * $P = 0.03274$ for 48h and ** $P = 0.001131$ for 72h). Source data are provided as a Source Data file.

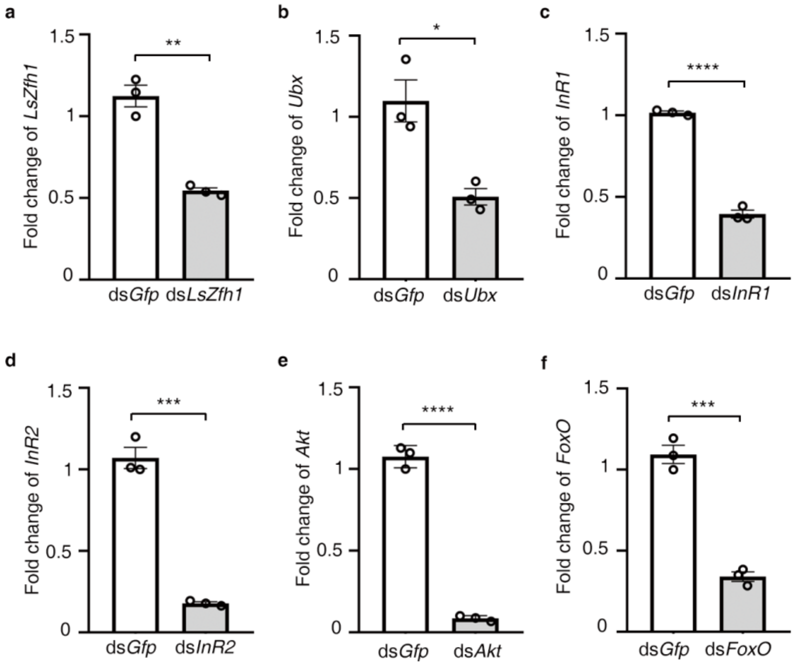

Supplementary Fig. 4. Examination of RNAi efficiency by qRT-PCR. Third-instar *Wt*^{SW} nymphs are microinjected with ds*LsZfh1*, ds*InR1*, ds*InR2*, ds*FoxO*, or ds*Gfp*. For RNAi-mediated knockdown of *Akt* and *Ubx*, fourth-instar *Wt*^{SW} nymphs were collected for microinjection. At two days after microinjection, insects (n = 5 for each of three replicates) were collected for RNA isolation. The relative expression of *LsZfh1* (**a**) is normalized to the expression of *rpl5*. The relative expression of *Ubx* (**b**), *InR1* (**c**), *InR2* (**d**), *Akt* (**e**), and *FoxO* (**f**) is normalized to the expression of *rps11*. The experiments were repeated three times with similar results (circles). Data are presented as mean ± SEM. Two-tailed unpaired *t*-test was used for the statistical analysis (**a**, ** P = 0.001067; **b**, * P = 0.01317; **c**, **** P = 1.82949E-05; **d**, *** P = 0.0001673; **e**, **** P = 1.62377E-05; **f**, *** P = 0.0002841). Source data are provided as a Source Data file.

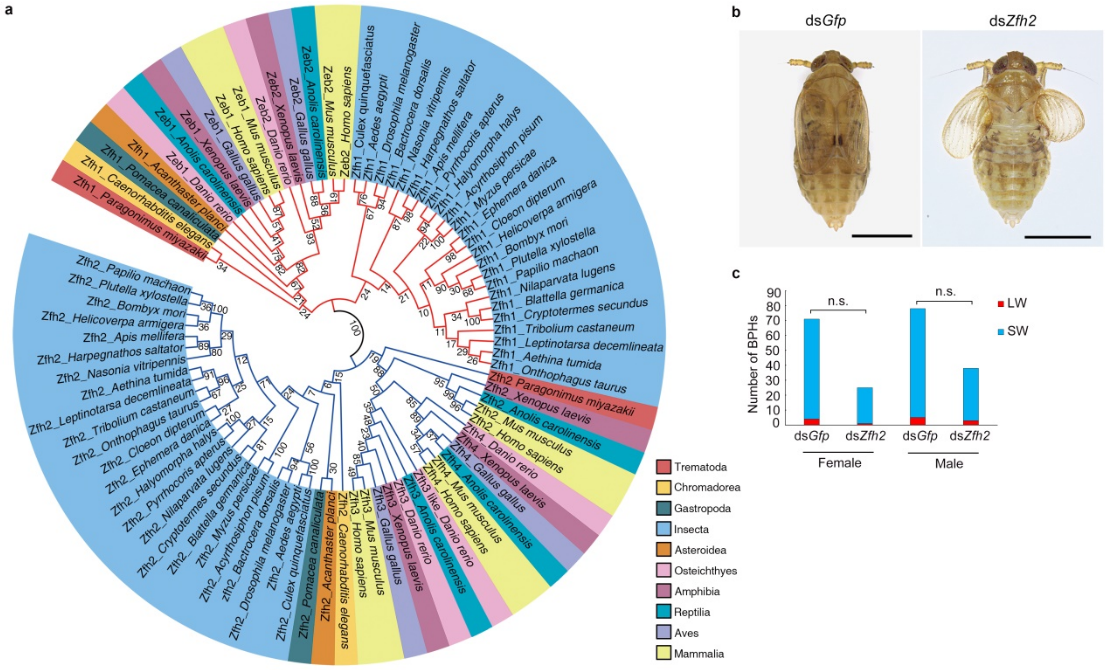

Supplementary Fig. 5. Phylogenetic analysis of Zfh1 and Zfh2. a A maximum-likelihood phylogenetic tree (bootstraps with 1000 replicates) is created using Zfh1 and Zfh2 protein sequences of different species from different classes (bottom right). The Zfh1 and Zfh2 branches are shown in red and blue lines, respectively. **b** Morphology of ds*Zfh2*- and ds*Gfp*-treated BPHs. Fourth-instar nymphs were microinjected with ds*Zfh2* or ds*Gfp*, and emerged adults were collected for morphological examination. Scale bars, 1mm. **c** Number of BPHs with different wing morphs upon ds*Zfh2* treatment. Fourth-instar nymphs were microinjected with ds*Zfh2* or ds*Gfp*, and emerged adults were collected for wing-morph counting. Two treatments are compared using Pearson χ^2 test (n.s., non-significant; $\chi^2 = 0.1$ and df = 1 for females, and $\chi^2 = 0.088$ and df = 1 for males). The experiment was repeated three times independently with similar results. SW, short-winged. LW, long-winged. Source data are provided as a Source Data file.

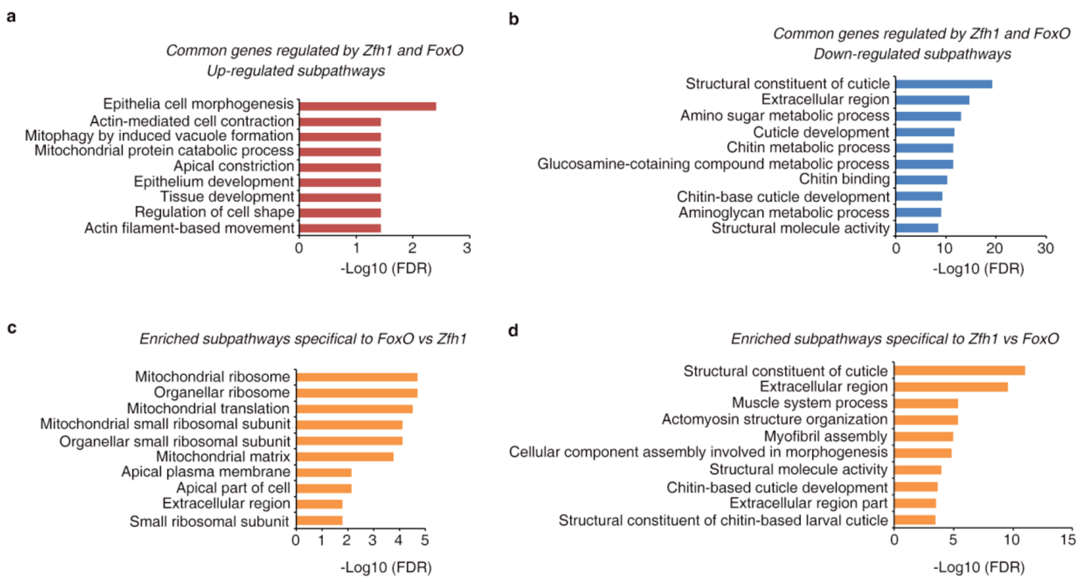

Supplementary Fig. 6. Gene ontology (GO) classification of differentially expressed genes. a GO terms enriched by common genes up-regulated by ds*zfh1* and ds*FoxO* relative to ds*Gfp*. **b** GO terms enriched by common genes down-regulated by ds*zfh1* and ds*FoxO* relative ds*Gfp*. **c** GO terms enriched by genes specifically regulated by ds*FoxO* versus ds*Zfh1*. **d** GO terms enriched by genes specifically regulated by ds*Zfh1* versus ds*FoxO*. Source data are provided as a Source Data file.

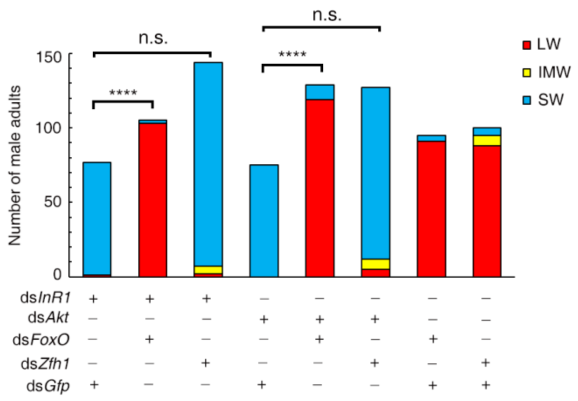

Supplementary Fig. 7. Number of males with different wing morphs upon double-gene knockdown. The LW ratio is compared between two groups using Pearson χ^2 test (ds*InR1*+ds*FoxO* vs ds*InR1*+ds*Gfp*: **** P = 7.5546E-39, χ^2 = 169.959, df = 1; ds*Akt*+ds*FoxO* vs ds*Akt*+ds*Gfp*: **** P = 5.4039E-38, χ^2 = 166.047, df = 1; n.s., not significant). Source data are provided as a Source Data file.

Supplementary Table 1. Mutagenesis rate of *FoxO* and *Zfh1* induced by CRISPR/Cas9.

Target	G0			G1
	Injected eggs	% (no.) hatched nymphs	% (no.) G0 adults with HA-tag	% (no.) germline transmission
FoxO	1023	13.2% (135/1023)	25% (11/44)	26.7% (32/120)
Zfh1	969	11.6% (112/969)	14.1% (9/64)	18.9% (17/90)
