# Supplementary material for: The transcription factor Zfh1 acts as a wing-morph switch in planthoppers
Source: Nat Commun. 2022 Sep 27;13:5670. doi: 10.1038/s41467-022-33422-6 (PMC9515195; doi:10.1038/s41467-022-33422-6)
Supplement: Supplementary file 3 — Description of Additional Supplementary Files [file 41467_2022_33422_MOESM3_ESM.pdf]

Description of Additional Supplementary Files

File Name: Supplementary Data 1
Description: Transcription factors differentially expressed between Wt^{LW} and Wt^{SW} fifth-instar nymphs.

File Name: Supplementary Data 2
Description: RNAi-mediated knockdown of transcription factor genes and wing morphs.

File Name: Supplementary Data 3
Description: BPH homeobox genes and RNAi phenotype.

File Name: Supplementary Data 4
Description: Differentially expressed genes between ds*Zfh1*- and ds*Gfp*-treated fifth-instar nymphs.

File Name: Supplementary Data 5
Description: Common genes regulated by ds*Zfh1*- and ds*FoxO*-treated fifth-instar nymphs.

File Name: Supplementary Data 6
Description: Primers used in this study.
