# Supplementary material for: The transcription factor Zfh1 acts as a wing-morph switch in planthoppers
Source: Nat Commun. 2022 Sep 27;13:5670. doi: 10.1038/s41467-022-33422-6 (PMC9515195; doi:10.1038/s41467-022-33422-6)
Supplement: Supplementary file 10 — Reporting Summary [file 41467_2022_33422_MOESM10_ESM.pdf]

nature portfolio

Corresponding author(s): Hai-Jun Xu

Last updated by author(s): Sep 2, 2022

Reporting Summary

Nature Portfolio wishes to improve the reproducibility of the work that we publish. This form provides structure for consistency and transparency in reporting. For further information on Nature Portfolio policies, see our Editorial Policies and the Editorial Policy Checklist.

Statistics

For all statistical analyses, confirm that the following items are present in the figure legend, table legend, main text, or Methods section.

n/a	Confirmed	
☐	☒	The exact sample size (*n*) for each experimental group/condition, given as a discrete number and unit of measurement
☐	☒	A statement on whether measurements were taken from distinct samples or whether the same sample was measured repeatedly
☐	☒	The statistical test(s) used AND whether they are one- or two-sided<br>*Only common tests should be described solely by name; describe more complex techniques in the Methods section.*
☐	☒	A description of all covariates tested
☐	☒	A description of any assumptions or corrections, such as tests of normality and adjustment for multiple comparisons
☐	☒	A full description of the statistical parameters including central tendency (e.g. means) or other basic estimates (e.g. regression coefficient) AND variation (e.g. standard deviation) or associated estimates of uncertainty (e.g. confidence intervals)
☐	☒	For null hypothesis testing, the test statistic (e.g. *F*, *t*, *r*) with confidence intervals, effect sizes, degrees of freedom and *P* value noted<br>*Give P values as exact values whenever suitable.*
☒	☐	For Bayesian analysis, information on the choice of priors and Markov chain Monte Carlo settings
☒	☐	For hierarchical and complex designs, identification of the appropriate level for tests and full reporting of outcomes
☒	☐	Estimates of effect sizes (e.g. Cohen's *d*, Pearson's *r*), indicating how they were calculated

Our web collection on statistics for biologists contains articles on many of the points above.

Software and code

Policy information about availability of computer code

Data collection	All qRT-PCR data was collected using CFX96 real-time PCR detection system (Bio-Rad). All RNAseq experiments were performed using Illumina Novaseq 6000 platform (Illumina). For Immunohistochemistry (IHC) staining, the confocal microscope Zeiss LSM 800 confocal microscopy (CarlZeiss MicroImaging) was used. For the observation of indirect flight muscle, JEM-1230 transmission electron microscope (JEOL) was used. For the Scanning electron microscope, SEM (TM-1000, Hitachi) was used. The Western blot results were detected using the Molecular Imager ChemiDoc XRS system (Bio-Rad). Images of wings and tibia were captured with a DFC320 digital camera attached to a Leica S8APO stereomicroscope using the LAS (v. 3.8) digital imaging system. Images of insects were taken using a DVM6 digital microscope (Leica Microsystems) with LAS X software.
Data analysis	The RNAseq data was analysed using Fastp (v0.12.4), hisat2 (v2.1.0), StringTie (v1.3.5), python script of prepDE.py (http://ccb.jhu.edu/software/stringtie/dl/prepDE.py), DESeq2 package (v1.36.0) and edgeR package (v. 3.38.4). Phylogenetic analysis was performed and plotted using BlastP (v2.2.31), MEGA-X (v10.1.8), iTOL tools (v6.5.8, https://itol.embl.de/), and Adobe Photoshop CC (v19.1.9). The wing size and hind tibia length were measured using ImageJ (v. 1.47). GO enrichment analysis were performed using OmicShare tool (https://www.omicshare.com/tools/home/report/goenrich.html). The heatmap was generated using the online OmicShare tool (https://www.omicshare.com/tools/Home/Soft/heatmap). Plotting and statistical analysis were performed with GraphPad Prism (v8.0.1).

For manuscripts utilizing custom algorithms or software that are central to the research but not yet described in published literature, software must be made available to editors and reviewers. We strongly encourage code deposition in a community repository (e.g. GitHub). See the Nature Portfolio guidelines for submitting code & software for further information.

Data

Policy information about availability of data

All manuscripts must include a data availability statement. This statement should provide the following information, where applicable:

- Accession codes, unique identifiers, or web links for publicly available datasets
- A description of any restrictions on data availability
- For clinical datasets or third party data, please ensure that the statement adheres to our policy

All data are available in the manuscript or the supplementary materials. NCBI accession numbers have been acquired for sequences generated in this study and are listed as follows: OM283826 (the Zfh1 gene, https://www.ncbi.nlm.nih.gov/nuccore/OM283826.1/), XP_039284941.1 (the Zfh2 gene, https://www.ncbi.nlm.nih.gov/protein/XP_039284941.1/), OM676634 (the LsZfh1 gene), PRJNA805393 (transcriptome, https://www.ncbi.nlm.nih.gov/bioproject/PRJNA805393), PRJNA805395 (transcriptome, https://www.ncbi.nlm.nih.gov/bioproject/?term=PRJNA805395), and PRJNA805400 (transcriptome, https://www.ncbi.nlm.nih.gov/bioproject/?term=PRJNA805400). Source data are provided with this paper.

Human research participants

Policy information about studies involving human research participants and Sex and Gender in Research.

Reporting on sex and gender	This is not relevant to my study.
Population characteristics	This is not relevant to my study.
Recruitment	This is not relevant to my study.
Ethics oversight	This is not relevant to my study.

Note that full information on the approval of the study protocol must also be provided in the manuscript.

Field-specific reporting

Please select the one below that is the best fit for your research. If you are not sure, read the appropriate sections before making your selection.

☒ Life sciences ☐ Behavioural & social sciences ☐ Ecological, evolutionary & environmental sciences

For a reference copy of the document with all sections, see nature.com/documents/nr-reporting-summary-flat.pdf

Life sciences study design

All studies must disclose on these points even when the disclosure is negative.

Sample size	No sample size calculation was performed. Sample size was determined from similar experiments in the literature and for all experiments a minimum of three biological replicates were analyzed per treatment or time-point.<br>For calculating wing morphs, more than 60 (n > 60) individuals for each sex were used. For isolation of total RNA for RNA sequencing, 20 individuals (n =20) were used. For measuring wing size and tibia length, 20 individuals (n = 20) were used. For RNAi efficiency examination, 5 individual (n = 5) for each three or five replicates were used. For determination of nymphal duration and survival rate, more than 10 individual (n > 10) were used. For determination of spatiotemporal expression of Zfh1, more than 15 individuals (n > 15) and 50 individual (n =50) were used for temporal- and spatio-expression, respectively. For western blot analysis, 40 individual (n= 40) and 35 nota (n = 35) were used.
Data exclusions	No data were excluded from the analyses.
Replication	All attempts at replication were successful.
Randomization	This is not relevant to my study. In this study, short-winged or long-winged morphs could be morphologically easily recognized following RNAi treatments.
Blinding	No blinding was conducted. This was not required as each experiment was designed to give an unambiguous outcome which did not depend on the judgement of the researchers.

Reporting for specific materials, systems and methods

We require information from authors about some types of materials, experimental systems and methods used in many studies. Here, indicate whether each material, system or method listed is relevant to your study. If you are not sure if a list item applies to your research, read the appropriate section before selecting a response.

Materials & experimental systems

n/a	Involved in the study
☐	☒ Antibodies
☐	☒ Eukaryotic cell lines
☒	☐ Palaeontology and archaeology
☐	☒ Animals and other organisms
☒	☐ Clinical data
☒	☐ Dual use research of concern

Methods

n/a	Involved in the study
☒	☐ ChIP-seq
☒	☐ Flow cytometry
☒	☐ MRI-based neuroimaging

Antibodies

Antibodies used	The horseradish peroxidase (HRP) conjugated goat anti-mouse antibody was provided by Proteintech (Cat#SA00001-1). The antibody against β-actin was provided by Huabio (Cat#M1210-2, Clone#B4-B2). The anti-HA monoclonal antibody was provided by MBL (Cat#M180-3, Clone#TANA2). The anti-6*His monoclonal antibody was provided by proteintech (Cat#66005-1-Ig, Clone#1B7G5).
Validation	All commercial antibodies were validated by the manufactures as indicated on their websites:<br>anti-6*His monoclonal antibody (Cat#66005-1-Ig, proteintech): https://www.ptgcn.com/products/His-Tag-Antibody-66005-1-Ig.htm<br>anti-β-actin mouse monoclonal antibody (Cat#M1210-2, Huabio): https://www.huabio.com/products/beta-actin-antibody-clone-a2-f6-monoclonal-m1210-2<br>anti-HA monoclonal antibody (Cat#M180-3): https://www.mblbio.com/bio/g/dtl/A/?pcd=M180-3

Eukaryotic cell lines

Policy information about cell lines and Sex and Gender in Research

Cell line source(s)	The HEK293T cell line was purchased from Pricella (Wuhan, China) and maintained in our laboratory.
Authentication	The cell line was STR tested.
Mycoplasma contamination	The cell line tested negative for mycoplasma contamination.
Commonly misidentified lines (See ICLAC register)	No commonly misidentified lines were used.

Animals and other research organisms

Policy information about studies involving animals; ARRIVE guidelines recommended for reporting animal research, and Sex and Gender in Research

Laboratory animals	The short-winged BPH strain was initially collected in Hangzhou, China, in 2008. The long-winged BPH strain was provided by Dr. Hong-Xia Hua (Huazhong Agricultural University, China). The planthopper (Laodelphax striatell) was collected in Hangzhou, China, 2018. All insects were maintained in Institute of Insect Sciences, Zhejiang university, China.
Wild animals	This is not relevant to my study.
Reporting on sex	This is not relevant to my study.
Field-collected samples	This is not relevant to my study.
Ethics oversight	Planthoppers were used in this study. No ethical approval or guidance was required.

Note that full information on the approval of the study protocol must also be provided in the manuscript.
